# Ca^2+^-induced changes in energy metabolism and viability of melanoma cells

**DOI:** 10.1038/sj.bjc.6690680

**Published:** 1999-09

**Authors:** L Glass-Marmor, J Penso, R Beitner

**Affiliations:** Health Sciences Research Center, Faculty of Life Sciences, Bar-Ilan University, Ramat Gan, 52900, Israel

**Keywords:** Melanoma, Ca^2+^, glycolysis, phosphofructokinase, hexokinase, glucose 1,6-bisphosphate

## Abstract

Cancer cells are characterized by a high rate of glycolysis, which is their primary energy source. We show here that a rise in intracellular-free calcium ion (Ca^2+^), induced by Ca^2+^-ionophore A23187, exerted a deleterious effect on glycolysis and viability of B16 melanoma cells. Ca^2+^-ionophore caused a dose-dependent detachment of phosphofructokinase (EC 2.7.1.11), one of the key enzymes of glycolysis, from cytoskeleton. It also induced a decrease in the levels of glucose 1,6-bisphosphate and fructose 1,6-bisphosphate, the two stimulatory signal molecules of glycolysis. All these changes occurred at lower concentrations of the drug than those required to induce a reduction in viability of melanoma cells. We also found that low concentrations of Ca^2+^-ionophore induced an increase in adenosine 5′-triphosphate (ATP), which most probably resulted from the increase in mitochondrial-bound hexokinase, which reflects a defence mechanism. This mechanism can no longer operate at high concentrations of the Ca^2+^-ionophore, which causes a decrease in mitochondrial and cytosolic hexokinase, leading to a drastic fall in ATP and melanoma cell death. The present results suggest that drugs which are capable of inducing accumulation of intracellular-free Ca^2+^ in melanoma cells would cause a reduction in energy-producing systems, leading to melanoma cell death. © 1999 Cancer Research Campaign


*British Journal of Cancer* (1999) **81**(2), 219–224
© 1999 Cancer Research Campaign
Article no. bjoc.1999.0680

### $Ca^{2+}$-induced changes in energy metabolism and viability of melanoma cells

**L Glass-Marmor, J Penso and R Beitner**

Health Sciences Research Center, Faculty of Life Sciences, Bar-Ilan University, Ramat Gan 52900, Israel

**Summary** Cancer cells are characterized by a high rate of glycolysis, which is their primary energy source. We show here that a rise in intracellular-free calcium ion ($Ca^{2+}$), induced by $Ca^{2+}$-ionophore A23187, exerted a deleterious effect on glycolysis and viability of B16 melanoma cells. $Ca^{2+}$-ionophore caused a dose-dependent detachment of phosphofructokinase (EC 2.7.1.11), one of the key enzymes of glycolysis, from cytoskeleton. It also induced a decrease in the levels of glucose 1,6-bisphosphate and fructose 1,6-bisphosphate, the two stimulatory signal molecules of glycolysis. All these changes occurred at lower concentrations of the drug than those required to induce a reduction in viability of melanoma cells. We also found that low concentrations of $Ca^{2+}$-ionophore induced an increase in adenosine 5′-triphosphate (ATP), which most probably resulted from the increase in mitochondrial-bound hexokinase, which reflects a defence mechanism. This mechanism can no longer operate at high concentrations of the $Ca^{2+}$-ionophore, which causes a decrease in mitochondrial and cytosolic hexokinase, leading to a drastic fall in ATP and melanoma cell death. The present results suggest that drugs which are capable of inducing accumulation of intracellular-free $Ca^{2+}$ in melanoma cells would cause a reduction in energy-producing systems, leading to melanoma cell death.

© 1999 Cancer Research Campaign

**Keywords**: Melanoma; $Ca^{2+}$; glycolysis; phosphofructokinase; hexokinase; glucose 1,6-bisphosphate

Cancer cells are characterized by a high rate of glycolysis, even under aerobic conditions, which is their primary energy source (Eigenbrodt et al, 1985; Fiechter and Gmünder, 1989; Beckner et al, 1990; Greiner et al, 1994). Glycolysis is controlled by allosteric regulators, among which glucose 1,6-bisphosphate (Glc-1,6-$P_2$) plays a major role in extrahepatic tissues (reviewed in Beitner, 1979, 1984, 1985, 1990), as well as by reversible binding of glycolytic enzymes to cytoskeleton (Arnold and Pette, 1968; reviewed in Clarke et al, 1985; Beitner, 1993; Pagliaro, 1993; Bereiter-Hahn et al, 1997). All glycolytic enzymes bind to cytoskeleton except hexokinase (EC 2.7.1.1), which binds reversibly to mitochondria, where it is linked to oxidative phosphorylation (Gots et al, 1972; Gots and Bessman, 1974; Viitanen et al, 1984; Wilson, 1985; Kottke et al, 1988; Adams et al, 1991). An increase in mitochondrially bound hexokinase was found in tumour cells and it has been suggested that this binding plays an important role in cancer cell metabolism (Arora and Pedersen, 1988; Fanciulli et al, 1994; reviewed in Golshani-Hebroni and Bessman, 1997).

We have previously found that an increase in intracellular-free calcium ion ($Ca^{2+}$) in normal cells, induced by $Ca^{2+}$-ionophore A23187, or $Ca^{2+}$-mobilizing hormones, exerts (through the $Ca^{2+}$–calmodulin complex), dual effects on cell energy metabolism (reviewed in Beitner, 1993, 1998). While a physiological increase in intracellular-free $Ca^{2+}$ increases mitochondrial-bound hexokinase and cytoskeleton-bound glycolytic enzymes, and thereby stimulates adenosine 5′-triphosphate (ATP) production, a high pathological rise in intracellular-free $Ca^{2+}$ causes a reduction in all the energy-producing systems in different cellular compartments, leading to cell death (Beery et al, 1980; Lilling and Beitner, 1990; Bassukevitz et al, 1992; Beitner and Lilling, 1993; Chen-Zion et al, 1993). Our experiments have also revealed that calmodulin antagonists are most effective drugs in the treatment of melanoma (Glass-Marmor et al, 1996; Glass-Marmor and Beitner, 1997; Beitner, 1998; Penso and Beitner, 1998).

In the present research we investigated how an increase in intracellular-free $Ca^{2+}$, induced by $Ca^{2+}$-ionophore A23187, would affect energy metabolism of melanoma cells. We studied its effect on cytoskeletal phosphofructokinase (PFK) (EC 2.7.1.11), and mitochondrially-bound hexokinase, the two key enzymes in glycolysis, and the levels of Glc-1,6-$P_2$, fructose 1,6-bisphosphate (Fru-1,6-$P_2$), ATP, and viability of the melanoma cells.

*Received 1 December 1998*
*Revised 17 March 1999*
*Accepted 24 March 1999*

*Correspondence to:* R Beitner

### MATERIALS AND METHODS

### Materials

$Ca^{2+}$-ionophore A23187 was obtained from Sigma Chemical Co. Other chemicals and enzymes were either from Sigma Chemical Co. or from Boehringer Mannheim GmbH. Tissue culture reagents were purchased from Biological Industries, Beit Haemek, Israel.

### Cell culture

B16 F10 mouse melanoma cells were grown in RPMI-1640 medium supplemented with 10% fetal calf serum and antibiotics, at 37°C in humidified atmosphere at 5% carbon dioxide and 95% air. Cells were passaged two to three times weekly.

This paper is part of the PhD thesis of LG-M to be submitted to the Senate of Bar-Ilan University, Ramat Gan, Israel.

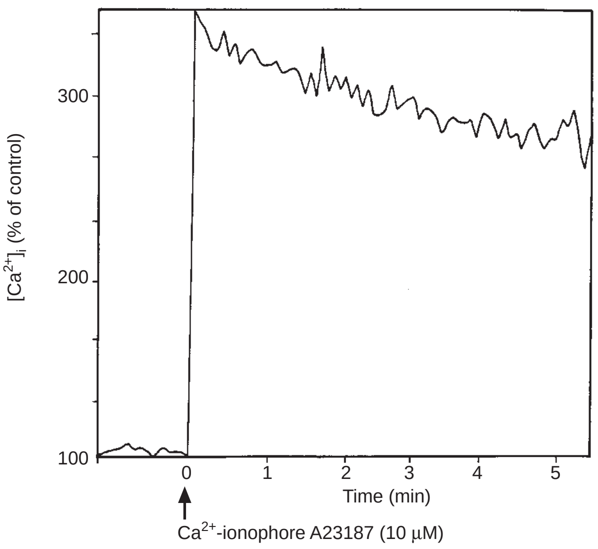

**Figure 1** Effect of $Ca^{2+}$-ionophore A23187 (10 µM) on intracellular free calcium, using Fura 2, in B16 melanoma cells. The resting level of intracellular free calcium was 240 ± 24 nM (*n* = 4). The results shown are a single representative experiment from six separate experiments

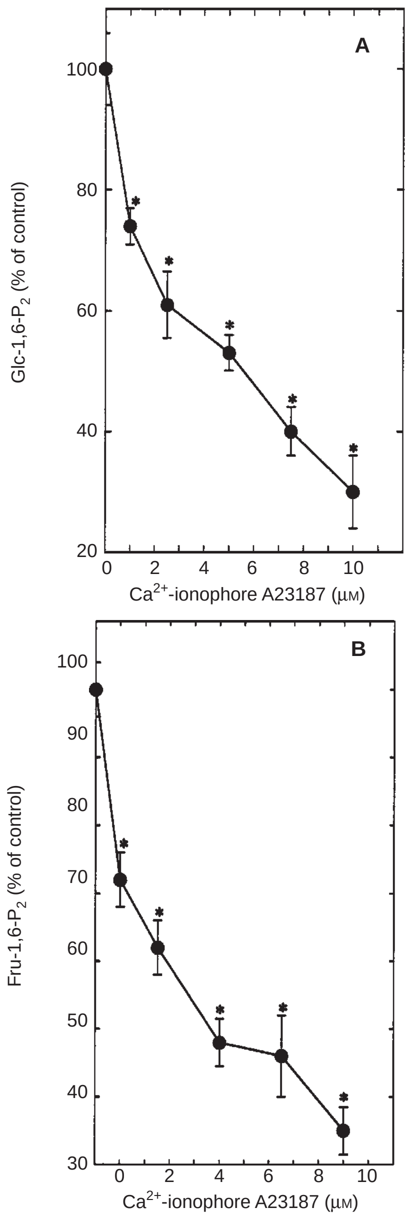

**Figure 2** Dose–response curves of the effect of $Ca^{2+}$-ionophore A23187 on glucose 1,6-bisphosphate (Glc-1,6-$P_2$) (**A**) and fructose 1,6-bisphosphate (Fru-1,6-$P_2$) (**B**) levels in B16 melanoma cells. Cells were incubated for 1 h in the absence and presence of different concentrations of $Ca^{2+}$-ionophore A23187. One hundred per cent Glc-1,6-$P_2$ and Fru-1,6-$P_2$ refers to 1.65 ± 0.09 and 14.5 ± 0.3 (nmol $mg^{-1}$ protein) respectively. Each point is the mean ± s.e.m. of 2–3 separate experiments which were performed in triplicate. *$P < 0.005$

### Treatment of culture

Melanoma cells ($8 \times 10^5$ cell $ml^{-1}$) were seeded in tissue culture plates (10 cm). After 48 h, cells were washed twice with phosphate-buffered saline (PBS). Then the cells were incubated at 37°C in PBS containing 5 mM glucose and 1.8 mM calcium chloride in the absence and presence of $Ca^{2+}$-ionophore A23187 for different concentrations. $Ca^{2+}$-ionophore A23187 was dissolved in dimethyl sulphoxide (DMSO). DMSO was added to the controls.

### Extraction and determination of glucose 1,6-bisphosphate, fructose 1,6-bisphosphate and ATP

Glucose 1,6-bisphosphate, fructose 1,6-bisphosphate and ATP were extracted from B16 melanoma cells, as described previously (Glass-Marmor et al, 1996). Glucose 1,6-bisphosphate was measured by the fluorometric method of Passonneau et al (1969); fructose 1,6-bisphosphate and ATP were measured by the method of Lowry et al (1964).

### Separation and assay of bound and soluble enzymes

Separation of cytoskeleton-bound and soluble phosphofructokinase from B16 melanoma cells was described previously (Glass-Marmor and Beitner, 1997). Cytoskeleton-bound and soluble phosphofructokinase were assayed as described previously (Lilling and Beitner, 1990).

Separation and assay of mitochondrial-bound and soluble hexokinase from B16 melanoma cells was described previously (Penso and Beitner, 1998).

### Measurement of intracellular free calcium

B16 melanoma cells were incubated with 4 µM Fura-2-AM for 40 min in balanced salt solution (BSS) (135 mM sodium chloride, 4.5 mM potassium chloride, 1.5 mM calcium chloride, 0.5 mM magnesium chloride, 5.6 mM glucose, 10 mM HEPES, pH 7.4, in double distilled water), at 37°C. Then the cells were harvested using trypsin (0.25%)–EDTA (0.05%) and precipitated by centrifugate at 270 ***g*** for 10 min. The cell suspension ($0.5 \times 10^6$ $ml^{-1}$), in BSS in 3-cm quartz cuvette, was excited at 335 nm with a slit 5-nm wide, and emissions collected at 500 nm at 30°C (Hill et al, 1989). Changes in fluorescence were recorded using a Perkin-Elmer LS-50 fluorescent spectrophotometer equipped with temperature-controlled stirred cuvette. To calibrate the fluorescence signal, we used 10 µM digitonin and 50 mM EGTA. Each sample was treated with 10 µM $Ca^{2+}$-ionophore A23187.

 © 1999 Cancer Research Campaign

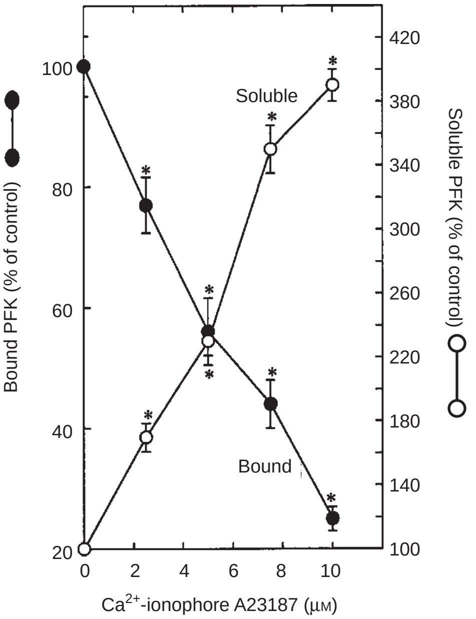

**Figure 3** Dose–response curves of the effect of $Ca^{2+}$-ionophore A23187 on cytoskeleton-bound and soluble phosphofructokinase (PFK) in B16 melanoma cells. Cells were incubated for 1 h in the absence and presence of different concentrations of $Ca^{2+}$-ionophore A23187. One hundred per cent activity of bound and soluble phosphofructokinase was 70 ± 5 and 14 ± 1 (mU $mg^{-1}$ protein) respectively. Each point is the mean ± s.e.m. of 2–3 separate experiments which were performed in triplicate. *$P < 0.005$

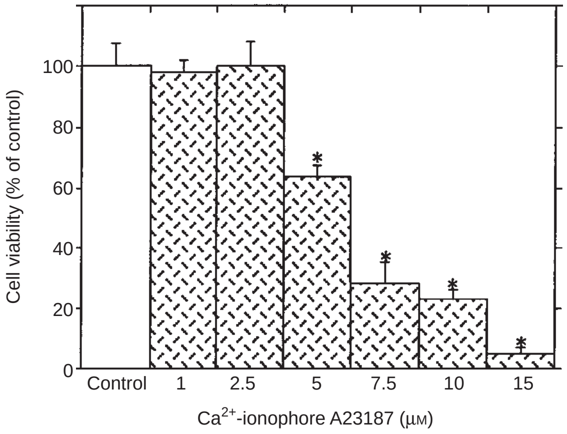

**Figure 4** Effect of $Ca^{2+}$-inophore A23187 on cell viability in B16 melanoma cells. Cells were incubated in the absence and presence of different concentrations of $Ca^{2+}$-ionophore A23187. One hundred per cent cell viability refers to $5 \times 10^6$ cells $ml^{-1}$. Values are the means ± s.e.m. of 2–3 separate experiments which were performed in triplicate. *$P < 0.005$

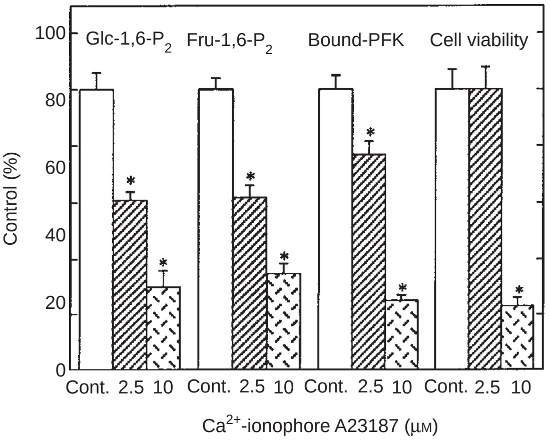

**Figure 5** Effect of $Ca^{2+}$-ionophore A23187 on cell viability and its relation to the levels of glucose 1,6-bisphosphate (Glc-1,6-$P_2$) and fructose 1,6-bisphosphate (Fru-1,6-$P_2$) and the binding of phosphofructokinase (PFK) to cytoskeleton in B16 melanoma cells. Cells were incubated with and without 2.5 μM and 10 μM $Ca^{2+}$-ionophore A23187 for 1 h. One hundred per cent cell viability refers to $5 \times 10^6$ cells $ml^{-1}$. One hundred per cent Glc-1,6-$P_2$ and Fru-1,6-$P_2$ levels refer to 1.65 ± 0.09 and 14.5 ± 0.3. One hundred per cent activity of bound PFK refers to 70 ± 5 (mU $mg^{-1}$ protein). Values are the mean ± s.e.m. of 2–3 separate experiments which were performed in triplicate. *$P < 0.005$

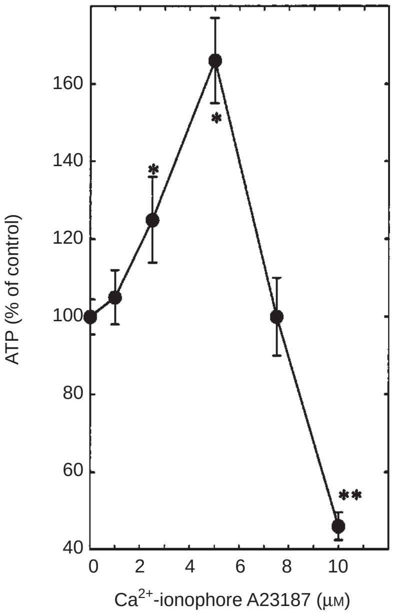

**Figure 6** Dose–response curves of the effect of $Ca^{2+}$-ionophore A23187 on ATP levels in B16 melanoma cells. Cells were incubated for 1 h in the absence and presence of different concentrations of $Ca^{2+}$-ionophore A23187. One hundred per cent ATP refers to 43.5 ± 2.1 (nmol $mg^{-1}$ protein). Each point is the mean ± s.e.m. of 2–3 separate experiments which were performed in triplicate. *$P < 0.05$, **$P < 0.005$

### Cell viability determination

After incubation in absence and presence of $Ca^{2+}$-ionophore A23187, the cells were harvested with trypsin (0.25%)–EDTA (0.05%) and centrifuged for 10 min at 270 ***g***. The precipitated cells were suspended in PBS and counted in a haemocytometer (Neubauer). Cell viability was determined by trypan blue dye exclusion.

### Protein measurement

Protein was measured by the method of Bradford (1976) with crystalline bovine serum albumin as a standard.

### RESULTS

The results presented in Figure 1 show that $Ca^{2+}$-ionophore A23187 induced an increase in intracellular concentration of free

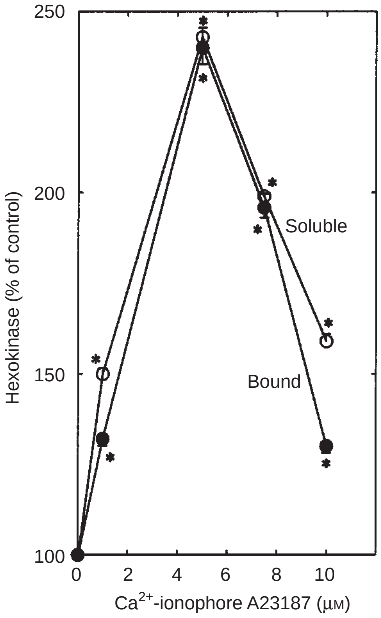

**Figure 7** Dose–response curves of the effect of $Ca^{2+}$-ionophore A23187 on mitochondrial-bound and soluble hexokinase in B16 melanoma cells. Cells were incubated for 1 h in absence and presence of different concentrations of $Ca^{2+}$-ionophore A23187. One hundred per cent activity of bound and soluble hexokinase was 8.97 ± 0.24 and 17.96 ± 0.41 (U $mg^{-1}$ protein) respectively. Each point is the mean ± s.e.m. of 2–3 separate experiments which were performed in triplicate. *$P < 0.005$

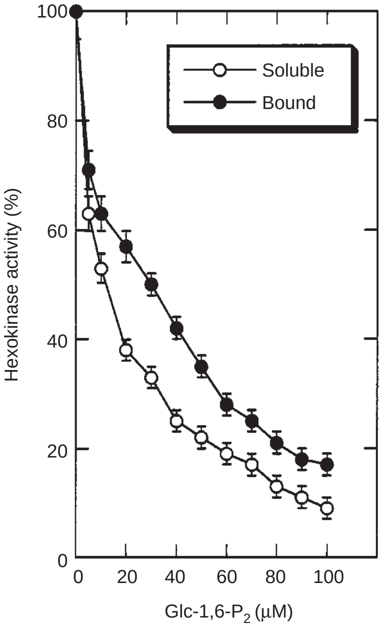

**Figure 8** The inhibitory effect of Glc-1,6-$P_2$ on the activity of mitochondrial-bound and soluble hexokinase in B16 melanoma cells. One hundred per cent activity of bound and soluble hexokinase was 3.44 ± 0.08 and 15.46 ± 0.32 (U $mg^{-1}$ protein) respectively. Each point is the mean ± s.e.m. of 4 separate experiments. $P < 0.005$ (each point vs control)

$Ca^{2+}$ in B16 melanoma cells. Figure 2 shows that $Ca^{2+}$-ionophore A23187 exerted a concentration-dependent decrease in the levels of Glc-1,6-$P_2$ (Figure 2A) and Fru-1,6-$P_2$ (Figure 2B) in melanoma cells.

The results presented in Figure 3 show that $Ca^{2+}$-ionophore A23187 exerted a dose-dependent decrease in cytoskeleton-bound phosphofructokinase in B16 melanoma cells, with a corresponding increase in soluble activity. Phosphofructokinase activity was assayed under maximal (optimal) conditions (pH 8.2), in which the enzyme is not sensitive to allosteric effectors (Beitner et al, 1978). Therefore, changes in the levels of allosteric regulators would not be expressed in its activity, and the results reveal solubilization of the cytoskeleton-bound enzyme.

The results presented in Figure 4 show that $Ca^{2+}$-ionophore A23187 induced a concentration-dependent reduction in viable melanoma cells. As shown in Figure 5, the decrease in Glc-1,6-$P_2$, Fru-1,6-$P_2$ and cytoskeleton-bound phosphofructokinase, occurred at lower concentrations of the drug than those required to decrease cell viability.

As shown in Figure 6, the level of ATP was elevated by low concentrations of $Ca^{2+}$-ionophore A23187, reaching maximum at 5 μM, and thereafter it was markedly reduced. The elevation in ATP resulted, most probably, from the concomitant increase in the activity of mitochondrial-bound hexokinase (Figure 7), which is linked to oxidative phosphorylation. A parallel increase in soluble hexokinase was also found (Figure 7). Hexokinase activity was assayed in these experiments under regulatory (suboptimal) conditions, in which it is sensitive to allosteric inhibition by Glc-1,6-$P_2$ (Beitner et al, 1979). As shown in Figure 8, the mitochondrial-bound and soluble hexokinase from B16 melanoma cells exhibited similar sensitivity to inhibition by Glc-1,6-$P_2$. When hexokinase activity was assayed under maximal (optimal) conditions, in which it is not sensitive to inhibition by Glc-1,6-$P_2$ (Beitner et al, 1979), no significant changes occurred in the activity of hexokinase from both the mitochondrial and soluble fractions of the cell; hexokinase maximal activity in the mitochondrial and soluble fractions of controls was 125.4 ± 1.8 and 472.4 ± 13.6 (U $mg^{-1}$ protein), respectively, and after treatment with 5 μM $Ca^{2+}$-ionophore A23187, it was 147.2 ± 4.4 and 466.8 ± 9.1 respectively.

### DISCUSSION

$Ca^{2+}$-ionophore A23187 induced an increase in intracellular-free $Ca^{2+}$ concentration in B16 melanoma cells (Figure 1). These results are compatible with the results of Hill et al (1989). The increase in intracellular-free $Ca^{2+}$ in melanoma cells induced by $Ca^{2+}$-ionophore A23187 caused solubilization of cytoskeleton-bound phosphofructokinase (Figure 3). The detachment of phosphofructokinase from cytoskeleton, would reduce cytoskeletal glycolysis and, thereby, the provision of local ATP, in the vicinity of the cytoskeleton membrane, and would affect cytoskeleton structure (Clarke et al, 1985).

The present results also reveal that $Ca^{2+}$-ionophore A23187 decreased the levels of Glc-1,6-$P_2$ (Figure 2A) and Fru-1,6-$P_2$ (Figure 2B), the two stimulatory signal molecules of glycolysis, which would also lead to a reduction of cytosolic glycolysis and ATP levels (Beitner, 1993). However, as shown in Figure 6, the levels of ATP were not reduced by low concentrations of $Ca^{2+}$-ionophore A23187, but rather elevated, reaching a maximum increase at a concentration of 5 μM, and thereafter decreased. The source of the increased ATP at low concentrations of the ionophore, is either mitochondrial or due to glycolysis. Mitochondrial-bound hexokinase was activated at low

© 1999 Cancer Research Campaign concentrations of the ionophore (Figure 7), which correlated with the increase in ATP (Figure 6), reaching a maximum at a concentration of 5 μM, and thereafter declined. Hexokinase was shown to bind to porin at the contact sites between the mitochondrial inner and outer membranes (Kottke et al, 1988; Adams et al, 1991; Brdiczka, 1991). The mitochondrially bound hexokinase preferentially utilizes mitochondrially-generated ATP (Gots et al, 1972; Gots and Bessman, 1974; Viitanen et al, 1984). In addition, the contacts were shown to have a higher $Ca^{2+}$-binding capacity compared to the outer and inner mitochondrial membrane. The mitochondrial-bound hexokinase enhances the uptake of $Ca^{2+}$ by the mitochondria (Kottke et al, 1988), and a rise in mitochondrial $Ca^{2+}$ stimulates intramitochondrial oxidative metabolism and ATP production (Denton and McCormack, 1990; McCormack and Denton, 1990).

The increase in mitochondrial-bound hexokinase was most probably not a result of translocation from the cytosol, as activity of the cytosolic (soluble) enzyme was also increased (Figure 7). The activation of both the mitochondrial-bound and soluble hexokinase resulted most probably from the decrease in Glc-1,6-$P_2$ levels (Figure 2A), which is a potent inhibitor of hexokinase from both the mitochondrial and soluble fractions of the melanoma cells (Figure 8). The findings that the activity of hexokinase was not changed under maximal (optimal) conditions, in which the enzyme is not subject to inhibition by Glc-1,6-$P_2$, strengthens this postulation. The increase in ATP induced by low concentrations of $Ca^{2+}$-ionophore, which most probably results from the increase in the mitochondrial-bound hexokinase activity, reflects a defence mechanism to prevent cell death. This mechanism can no longer operate at high concentrations of the $Ca^{2+}$-ionophore, which causes a decrease in mitochondrial-bound hexokinase (Figure 7), leading to a fall in ATP (Figure 6) and cell death (Figure 4). These experiments fit with the general schemes of apoptosis following accumulation of intracellular free calcium, as described recently by Ichas and Mazat (1998). The increase in cytosolic calcium can induce an increase in mitochondrial calcium, leading to an opening of the permeability transition pore, accompanied by a drop in mitochondrial transmembrane potential.

In contrast to hexokinase, the activity of cytoskeleton-bound phosphofructokinase was not increased by low concentrations of $Ca^{2+}$-ionophore but rather markedly reduced (Figure 3). This differs from normal tissues, in which we found a dual effect of $Ca^{2+}$ on cytoskeleton-bound glycolytic enzymes (Beitner, 1993, 1998). This may be due to the changes in cytoskeleton structure and function in cancer cells (Rao and Cohen, 1991).

The $Ca^{2+}$-induced decrease in the levels of Glc-1,6-$P_2$ and Fru-1,6-$P_2$ and the detachment of phosphofructokinase from cytoskeleton, occurred at lower concentrations of the drug than the reduction in cell viability (Figure 5), which indicates that these are primary changes which lead to cell death.

In summary, the present results reveal that accumulation of high concentrations of intracellular free $Ca^{2+}$ in melanoma cells, induces a reduction in the energy-producing systems in different cellular compartments, leading to melanoma cell death. This may be the mechanism of action of certain drugs which are already used for melanoma treatment, and may also serve to evaluate the therapeutic action of new drugs.

### ACKNOWLEDGEMENTS

The skillful technical assistance of Mrs H Morgenstern and Mrs H Ben-Porat is acknowledged with thanks. This work was supported in part by the ALSAM Foundation, Los Angeles, CA, USA, and by the Health Sciences Research Center and the Research Committee, Bar-Ilan University, Israel.

### REFERENCES

Adams V, Griffin L, Towbin J, Gelb B, Worley K and McCabe ERB (1991) Porin interaction with hexokinase and glycerol kinase: metabolic microcompartmentation at the outer mitochondrial membrane. *Biochem Med Metab Biol* **45**: 271–291

Arnold H and Pette D (1968) Binding of glycolytic enzymes to structure proteins of the muscle. *Eur J Biochem* **6**: 163–171

Arora KK and Pedersen PL (1988) Functional significance of mitochondrial-bound hexokinase in tumor cell metabolism. *J Biol Chem* **263**: 17422–17428

Bassukevitz Y, Chen-Zion M and Beitner R (1992) $Ca^{2+}$-ionophore A23187 and the $Ca^{2+}$-mobilizing hormones serotonin, vasopressin, and bradykinin increase mitochondrially bound hexokinase in muscle. *Biochem Med Metab Biol* **47**: 181–188

Beckner ME, Stracke ML, Liotta LA and Schiffmann E (1990) Glycolysis as primary energy source in tumor cell chemotaxis. *J Natl Cancer Inst* **82**: 1836–1840

Beery E, Klein S, Nordenberg J and Beitner R (1980) The influence of $Ca^{2+}$-ionophore A23187 on glucose-1,6-diphosphate and ATP levels, and on the activities of phosphofructokinase and phosphoglucomutase, in isolated rat diaphragm muscle. *Biochem Int* **1**: 526–531

Beitner R (1979) The role of glucose-1,6-diphosphate in the regulation of carbohydrate metabolism in muscle. *Trends Biochem Sci* **4**: 228–230

Beitner R (1984) Control of levels of glucose 1,6-bisphosphate. *Int J Biochem* **16**: 579–585

Beitner R (1985) Glucose 1,6-bisphosphate – the regulator of carbohydrate metabolism. In: *Regulation of Carbohydrate Metabolism*, Beitner R (ed), Vol I, pp 1–27. CRC Press: Boca Raton, Florida

Beitner R (1990) Regulation of carbohydrate metabolism by glucose-1,6-bisphosphate in extrahepatic tissues; comparison with fructose-2, 6-bisphosphate. *Int J Biochem* **22**: 553–557

Beitner R (1993) Control of glycolytic enzymes through binding to cell structures and by glucose 1,6-bisphosphate under different conditions. The role of $Ca^{2+}$ and calmodulin. *Int J Biochem* **25**: 297–305

Beitner R (1998) Calmodulin antagonists and cell energy metabolism in health and disease. *Mol Genet Metab* **64**: 161–168

Beitner R and Lilling G (1993) Treatment of muscle damage, induced by high intracellular $Ca^{2+}$, with calmodulin antagonists. *Gen Pharmacol* **24**: 847–855

Beitner R, Haberman S, Nordenberg J and Cohen TJ (1978) The levels of cyclic GMP and glucose-1,6-diphosphate, and the activity of phosphofructokinase, in muscle from normal and dystrophic mice. *Biochim Biophys Acta* **542**: 537–541

Beitner R, Nordenberg J and Cohen TJ (1979) Correlation between the levels of glucose-1,6-diphosphate and the activities of phosphofructokinase, phosphoglucomutase and hexokinase, in skeletal and heart muscles from rats of different ages. *Int J Biochem* **10**: 603–608

Bereiter-Hahn J, Airas J and Blum S (1997) Supramolecular associations with the cytomatrix and their relevance in metabolic control: protein synthesis and glycolysis. *Zoology* **100**: 1–24

Bradford MM (1976) A rapid and sensitive method for the quantitation of microgram quantities of protein utilizing the principle of protein dye binding. *Anal Biochem* **72**: 248–254

Brdiczka D (1991) Contact sites between mitochondrial envelope membranes. Structure and function in energy and protein-transfer. *Biochim Biophys Acta* **1071**: 291–312

Chen-Zion M, Lilling G and Beitner R (1993) The dual effects of $Ca^{2+}$ on binding of the glycolytic enzymes, phosphofructokinase and aldolase, to muscle cytoskeleton. *Biochem Med Metab Biol* **49**: 173–181

Clarke F, Stephan P, Morton D and Weidemann J (1985) Glycolytic enzyme organization via the cytoskeleton and its role in metabolic regulation. In: *Regulation of Carbohydrate Metabolism*, Beitner R (ed), Vol II, pp 1–31. CRC Press: Boca Raton, Florida

Denton RM and McCormack JG (1990) $Ca^{2+}$ as a second messenger within mitochondria of the heart and other tissues. *Annu Rev Physiol* **52**: 451–466

Eigenbrodt E, Fister P and Reinacher M (1985) New perspectives on carbohydrate metabolism in tumor cells. In *Regulation of Carbohydrate Metabolism*, Beitner R (ed), Vol II, pp 141–179. CRC Press: Boca Raton, Florida

Fanciulli M, Paggi MG, Bruno T, DelCarlo C, Bonetto F, Gentile FP and Floridi A (1994) Glycolysis and growth rate in normal and in hexokinase-transfected NIH/3T3 cells. *Oncol Res* **6**: 405–409

© 1999 Cancer Research Campaign 

Fiechter A and Gmünder FK (1989) Metabolic control of glucose degradation in yeast and tumor cells. *Adv Biochem Eng Biotechnol* **39**: 1–28

Glass-Marmor L and Beitner R (1997) Detachment of glycolytic enzymes from cytoskeleton of melanoma cells induced by calmodulin antagonists. *Eur J Pharmacol* **328**: 241–248

Glass-Marmor L, Morgenstern H and Beitner R (1996) Calmodulin antagonists decrease glucose 1,6-bisphosphate, fructose 1,6-bisphosphate, ATP and viability of melanoma cells. *Eur J Pharmacol* **313**: 265–271

Golshani-Hebroni SG and Bessman SP (1997) Hexokinase binding to mitochondria: a basis for proliferative energy metabolism. *J Bioenerg Biomembr* **29**: 331–338

Gots RE and Bessman SP (1974) The functional compartmentation of mitochondrial hexokinase. *Arch Biochem Biophys* **163**: 7–14

Gots RE, Gorin FA and Bessman SP (1972) Kinetic enhancement of bound hexokinase activity by mitochondrial respiration. *Biochem Biophys Res Commun* **49**: 1249–1255

Greiner EF, Guppy M and Brand K (1994) Glucose is essential for proliferation and the glycolytic enzyme induction that provokes a transition to glycolytic energy production. *J Biol Chem* **269**: 31484–31490

Hill SE, Bleehen SS and MacNeil S (1989) 1α-25-dihydroxyvitamin $D_3$ increases intracellular free calcium in murine $B_{16}$ melanoma. *Br J Dermatol* **120**: 21–30

Ichas F and Mazat J-P (1998) From calcium signaling to cell death: two conformations for the mitochondrial permeability transition pore. Switching from low- to high-conductance state. *Biochim Biophys Acta* **1366**: 33–50

Kottke M, Adams V, Riesinger I, Bremm G, Bosch W, Brdiczka D, Sandri G and Panfili E (1988) Mitochondrial boundary membrane contact sites in brain: points of hexokinase and creatine kinase location, and control of $Ca^{2+}$ transport. *Biochim Biophys Acta* **935**: 87–102

Lilling G and Beitner R (1990) Decrease in cytoskeleton-bound phosphofructokinase in muscle induced by high intracellular calcium, serotonin and phospholipase $A_2$ *in vivo. Int J Biochem* **22**: 857–863

Lowry OH, Passonneau JV, Hasselberger FX and Schulz DW (1964) Effect of ischemia on known substrates and cofactors of the glycolytic pathway in brain. *J Biol Chem* **239**: 18–30

McCormack JG and Denton RM (1990) The role of mitochondrial $Ca^{2+}$ transport and matrix $Ca^{2+}$ in signal transduction in mammalian tissues. *Biochim Biophys Acta* **1018**: 287–291

Pagliaro L (1993) Glycolysis revisited – a funny thing happened on the way to the Krebs cycle. *NIPS* **8**: 219–223

Passonneau JV, Lowry OH, Schulz DW and Brown JG (1969) Glucose 1,6-diphosphate formation by phosphoglucomutase in mammalian tissues. *J Biol Chem* **244**: 902–909

Penso J and Beitner R (1998) Clotrimazole and bifonazole detach hexokinase from mitochondria of melanoma cells. *Eur J Pharmacol* **342**: 113–117

Rao KMK and Cohen HJ (1991) Actin cytoskeletal network in aging and cancer. *Mutation Res* **256**: 139–148

Viitanen PV, Geiger PJ, Erickson-Viitanen S and Bessman SP (1984) Evidence for functional hexokinase compartmentation in rat skeletal muscle mitochondria. *J Biol Chem* **259**: 9679–9684

Wilson JE (1985) Regulation of mammalian hexokinase activity. In: *Regulation of Carbohydrate Metabolism*, Beitner R (ed), Vol I, pp. 45–85. CRC Press: Boca Raton, Florida

 *© 1999 Cancer Research Campaign*
